# Intussusception in an elderly female and its management: A case report and literature review

**DOI:** 10.1002/ccr3.7745

**Published:** 2023-08-10

**Authors:** Lokeshwar Raaju Addi Palle, Pugazhendi Inban, Ogbonnaya Akuma, Chinaza Mercy Akuma, Hema Anisha Srirama, Keval Thakkar, Ihab Sheikh Hanafi, Rojaj Dahal, Mohammed Adnaan Yousuf, Aadil Mahmood Khan, Anasonye Emmanuel Kelechi

**Affiliations:** ^1^ Madha Medical College and Hospital Chennai India; ^2^ Government Medical College Omandura Chennai India; ^3^ Ebonyi State University Abakaliki Nigeria; ^4^ Chamberlain University College of Health Professions Chicago Illinois USA; ^5^ Narayana Medical College and Hospital Nellore India; ^6^ Georgetown University Washington DC USA; ^7^ Spartan Health Sciences University Vieux Fort Saint Lucia; ^8^ Manipal College of Medical Sciences (MCOMS) Pokhara Nepal; ^9^ Chicago Clinical Research Institute Chicago Illinois USA; ^10^ University of Illinois Chicago Chicago Illinois USA; ^11^ Texila American University Georgetown Guyana

**Keywords:** computed tomography (CT) scans, gastrointestinal endoscopy, GJ tube, intussusception, mesenteric vessels

## Abstract

Intussusception is the telescoping or invagination of the proximal part of the gastrointestinal tract into an adjacent section. It is rare in adults, accounting for 1% of adult bowel obstruction. Adult presentation of intussusception is variable, with nonspecific, vague symptoms like abdominal pain, nausea, vomiting, and rectal bleeding. Abdominal computed tomography (CT) scans have the highest sensitivity in the diagnosis of intussusception. The classical findings of intussusception are the target sign and mesenteric vessels lined within the bowel lumen. An abdominal CT scan can reveal a cloverleaf figuration, fluid‐filled ileal loops, superior mesenteric venous (SMV) occlusion, and concerns about ongoing sealed perforation or fistulization. Our patient is an 86‐year‐old female who was diagnosed with a jejunal–jejunal long‐segment intussusception, gastro‐enteric fistula, and SMV occlusion with distal reconstitution. The patient responded well to conservative treatment and was discharged for follow‐up.

## 1 INTRODUCTION

Intussusception is a condition that occurs when a segment of the intestine folds inward into an adjacent segment, leading to an obstruction and eventually ischemia. It has been considered a pediatric condition, but recent literature has shown that intussusception can also affect adults, including the elderly.^1^, ^2^ However, there is a paucity of literature on intussusception in the elderly.

Intussusception is a serious condition that can lead to significant morbidity and mortality if not treated promptly. The etiology and management of intussusception are not well defined, especially in the elderly population. Adult intussusception is varied, unlike in children. Adults with intussusception frequently have nonspecific symptoms such as stomach discomfort, nausea, vomiting, and rectal bleeding.^3^ The diagnosis of intussusception in adults is typically made with computed tomography (CT).

We report a case of an 86‐year‐old female patient who presented with a history of abdominal pain and distension. After a thorough investigation, she was diagnosed with intussusception, which was successfully managed conservatively. This case highlights the importance of early diagnosis and prompt management of intussusception in the elderly population.

## 2 CASE PRESENTATION

We present the case of an 86‐year‐old female patient who came to the emergency department with a chief complaint of abdominal pain and intermittent leaking from a gastro‐jejunum (GJ) tube for a month duration. Her past medical history includes advanced Parkinson's disease, memory impairment, limb dystonia, hypertension, provoked pulmonary embolism, chronic constipation, and gastrojejunostomy. The patient originally had a percutaneous endoscopically placed GJ tube placed for administration of carbidopa/levodopa for Parkinson's disease. Due to obstruction, she had to have this revised later in the year. Following this revision, she began having intermittent cramping, abdominal pain, and leakage from the GJ tube site. At the time, she had a CT of the abdomen and pelvis that showed no obstruction or significant abnormalities. Symptoms improved somewhat; however, 6–8 months later, she felt the symptoms returned, and also noted that her GJ tube was leaking and the stoma was irritated. Her abdominal pain at the time was significant enough to require narcotics and epidural pain treatment.

**FIGURE 1:**
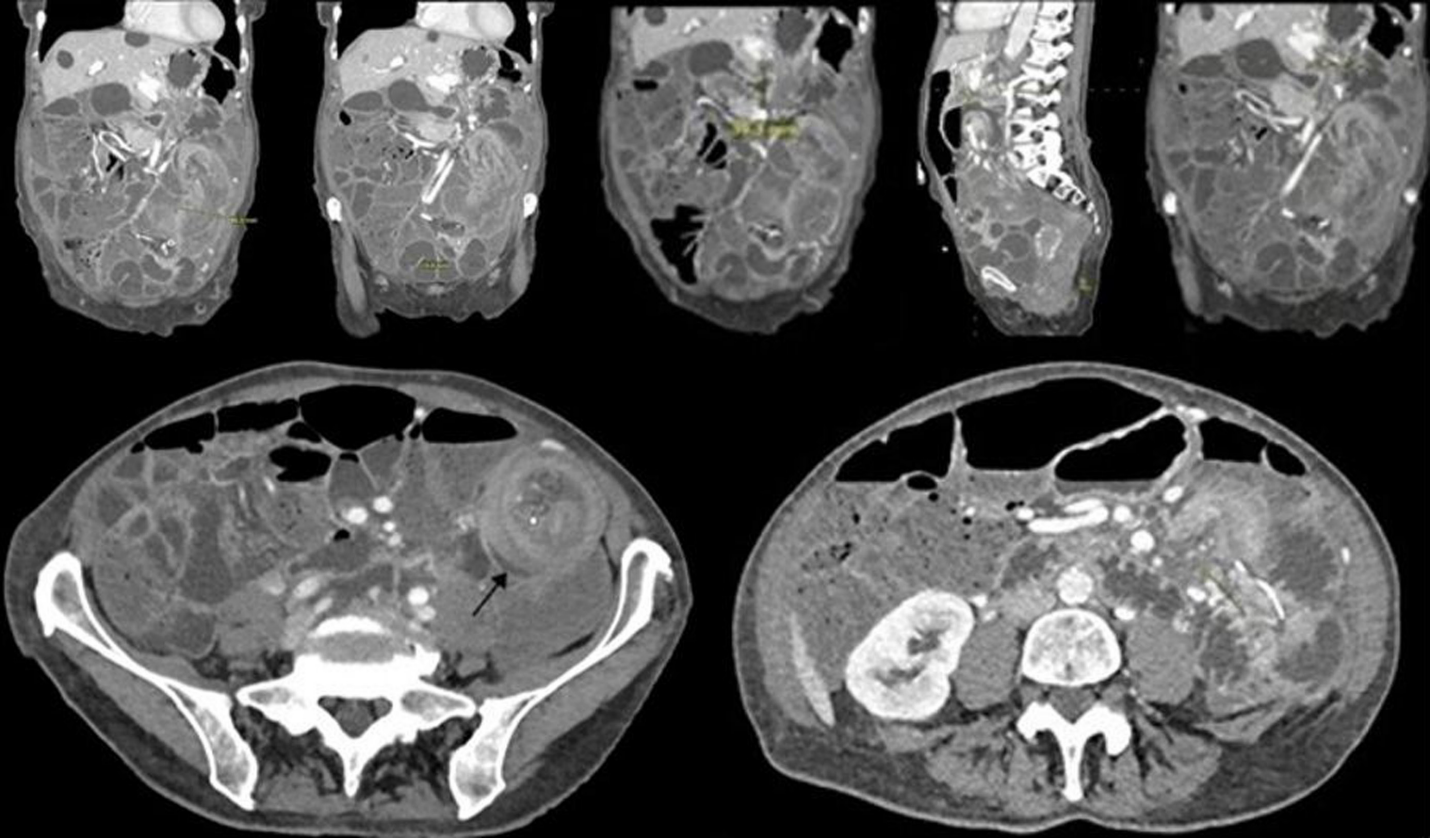
CT scan showing the jejunostomy limb with new long‐segment jejunal–jejunal intussusception (arrow) and associated small bowel obstruction.

Due to the pain, a repeat CT was done, which showed a cloverleaf figuration showing the GJ tube bypassing the duodenum and leading into the jejunum, raising concern for ongoing sealed perforation or fistulization and a GJ tube with an intraluminal anchor applied to the gastric wall. Additionally, a small bowel obstruction involving the long‐segment jejunal–jejunal intussusception was associated with the jejunostomy limb of the GJ tube (Figure 1). The CT also showed superior mesenteric venous occlusion (SMV), which was not present in previous imaging. Throughout this time, the patient has had Parkinson's medications actively managed and chronic constipation and electrocardiogram abnormalities managed by a respective specialist team.

On presentation to the emergency room (ER), she said she is feeling fine. Her abdominal pain was not there. She has a normal appetite, but has not eaten recently. Her bowel habits have not changed from roughly once every other day of soft and not fully formed stool. She denied nausea, vomiting, headache, chest pain, and shortness of breath. She denied any urinary changes in frequency, color, or smell. In addition, her labs were normal.

Imaging for chronic abdominal pain since the exchange of the GJ tube demonstrated a likely gastro‐enteric fistula, jejunal–jejunal long‐segment intussusception, and SMV occlusion with distal reconstitution. Surgery was consulted by gastroenterology for assistance with the management of jejunal–jejunal intussusception, likely caused by the jejunal portion of the GJ tube acting as a lead point. No acute surgical intervention was required for intussusception as the patient was without signs of bowel ischemia on imaging or laboratories, had normal vital signs, had chronic abdominal symptoms without any new complaints, and was not peritonitic on examination because of the absence of worsening abdominal pain or tenderness, new‐onset nausea and emesis/obstructive symptoms, tachycardia, and hypotension.

The plan was made to remove the jejunostomy portion of the GJ tube endoscopically, and the patient was nil per oral (NPO). The patient responded well to the treatment given and was discharged for follow‐up.

## 3 DISCUSSION

Intussusception is the telescoping or invagination of the proximal part of the gastrointestinal tract into an adjacent section.^4^ Intussusception predominantly occurs in childhood and is rare in adults, with an approximate incidence of 2–3 per 1,000,000 per year, and accounting for 1% of all adult causes of bowel obstruction.^2^, ^5^ Adult intussusception constitutes <5% of all cases of intussusception.^6^ Adult presentation of intussusception is variable, which is unlike the intussusception that presents in children. Symptoms may be acute or chronic. Intussusception in adults is not easily diagnosed, because patients usually present with nonspecific vague symptoms and not consistent with an acute abdomen. The symptoms include abdominal pain, nausea, vomiting, and rectal bleeding.^3^

About 70%–90% of adult intussusception is secondary to an underlying pathology whereas approximately 90% of intussusception in children is idiopathic.^7^, ^8^, ^9^ Additionally, 52%–55% of adult intussusception cases occur in the distal portion of the small bowel and 38%–45% occur in the large intestine.^10^ Colo‐colic is the most common adult type of intussusception.^11^ Other rare locations are gastroduodenal and jejunogastric intussusception that follows gastro‐jejunostomy.

Gastroduodenal intussusception accounts for only 10% of documented adult intussusception.^12^ Our patient was a jejunal–jejunal intussusception which is rare for adult as well. The mean age of adult intussusception is 50 years, with an almost equal male‐to‐female ratio.^11^ Our patient was an 86 years old, making it a rare finding of intussusception in this age group when compared to other cases.

Abdominal CT scan has the highest sensitivity in the diagnosis of intussusception with 58%–100%.^13^ The classical findings show the target sign or the sausage‐shaped soft tissue mass with a layering effect. Mesenteric vessels lined within the bowel lumen are also described typically.^14^ The abdominal CT done for our patient revealed a cloverleaf figuration, more pronounced than her original CT. The ileal loops were fluid‐filled, and the colon was distended with air, fluid, and stool. Moreover, SMV occlusion was not present when the first abdominal CT scan was done. There were also concerns about ongoing sealed perforation and fistulization, possibly from the bypassing of the duodenum by the GJ tube.

The CT scan can differentiate between the lead point and non‐lead point intussusceptions. In cases of sub‐acute or chronic large bowel obstruction, the flexible lower gastrointestinal endoscopy is vital in confirming the diagnosis of intussusception, determining its location, and finding the underlying lesion which may serve as an option for treatment.^14^

Although adult intussusception is almost always managed surgically, and there are debates about whether intussusception should be reduced before resection, our patient's management was informed by the assessment of jejunal–jejunal long‐segment intussusception and gastro‐enteric fistula, SMV occlusion with distal reconstitution. This diagnosis was made after a careful review of both the clinical presentation and the findings from the CT scan. No urgent surgical intervention was considered because the patient showed no sign of bowel ischemia on imaging and had normal laboratory findings and this is quite dissimilar to the traditional surgical interventions that are done in intussusception cases, which made our case scenario a rare type. There were also no new clinical findings apart from the pre‐existing chronic abdominal pain. The patient was placed in NPO and to plan was to remove the jejunostomy portion of the GJ tube endoscopically.

Intussusception itself has a good prognosis; however, the main prognostic factor that typically affects the course of the disease comes from the nature of the causative lesion.^14^ Our patient responded well to treatment and was discharged for follow‐up.

## 4 CONCLUSION

This case report depicts a rare instance of intussusception in an elderly female. Since there is no specific etiology when it comes to adults, it is important to consider intussusception as an early differential diagnosis. Intussusception is always predominantly managed surgically; however, it also depends on the clinical symptoms. Since this patient did not have the typical signs and symptoms of intussusception, there were no immediate surgical interventions performed. However, later on, the diagnosis of intussusception was confirmed and the patient responded well to the treatment given. This report adds to the rare literature available on the management of intussusception in an elderly group. It also spreads awareness to remind clinicians to keep intussusception as a differential diagnosis when an elderly patient presents with vague abdominal symptoms. Prompt diagnosis and early management are always key in this age group to prevent mortality.

## AUTHOR CONTRIBUTIONS

**Lokeshwar Raaju Addi Palle:** Supervision; writing – original draft. **Pugazhendi Inban:** Writing – original draft; writing – review and editing. **Ogbonnaya Akuma:** Writing – review and editing. **Chinaza Mercy Akuma:** Resources; software. **Hema Anisha Srirama:** Conceptualization; writing – original draft; writing – review and editing. **Keval Thakkar:** Resources; software; writing – original draft. **Ihab Sheikh Hanafi:** Writing – original draft; writing – review and editing. **Rojaj Dahal:** Supervision; validation. **Mohammed Adnaan Yousuf:** Visualization; writing – original draft. **Aadil Khan:** Software; supervision; validation; writing – original draft; writing – review and editing. **Anasonye Emmnauel Kelechi:** Conceptualization; software.

## FUNDING INFORMATION

None.

## CONFLICT OF INTEREST STATEMENT

None.

## CONSENT

Consent statement should be added to all articles: "Written informed consent was obtained from the patient to publish this report in accordance with the journal's patient consent policy.”

## Data Availability

The datasets analyzed during the current study are available from the corresponding author upon reasonable request. Additionally, comprehensive literature sources used for the literature review are cited appropriately within the manuscript.
